# ChatGPT guidance for reproductive specialists: Dr. Jekyll or Mr. Hyde?

**DOI:** 10.17179/excli2023-6120

**Published:** 2023-08-29

**Authors:** Pallav Sengupta, Sulagna Dutta

**Affiliations:** 1 Department of Biomedical Sciences, College of Medicine, Gulf Medical University, Ajman, UAE; 2 School of Medical Sciences, Bharath Institute of Higher Education and Research (BIHER), Tamil Nadu, India

## ⁯

### Rise of ChatGPT in reproductive medicine

ChatGPT is a cutting-edge series of machine learning models designed by OpenAI. Built on the Transformer architecture, these models make use of deep learning techniques to comprehend and create text that resembles human-like language. They are trained on extensive collections of text data and use a multi-layered mechanism that recognizes the sequential character of language. This understanding facilitates the generation of text that is contextually pertinent and coherent. With applications that span various natural language processing tasks, including text completion, translation, and summarization, ChatGPT helps to narrow the divide between human and machine communication.

The expansion of ChatGPT applications within healthcare signals a significant transformation, especially in the field of reproductive medicine. This emerging technology embodies the intersection between artificial intelligence (AI) and medical expertise, leading to a paradigm shift that reshapes both patient interaction and the delivery of care **(**Supplementary Figure 1**)**. Through the utilization of the analytical capabilities of ChatGPT, medical professionals can obtain unprecedented insights into complex reproductive issues, thereby accelerating the development of individualized fertility treatments. Furthermore, the skilful abilities of ChatGPT to process and analyze vast amounts of medical data enables the extraction of pertinent information. This enhances effective communication between patient and practitioner, ultimately contributing to the advancement of reproductive healthcare.

This correspondence is intended to stimulate discussion and offer insights into the growing application of ChatGPT, a complex linguistic model built on the GPT-4 architecture, within the sphere of reproductive medicine. Specifically, our objective is to explore the potential advantages and disadvantages associated with the implementation of such an AI system, as well as to discuss the ethical dilemmas it may present.

### ChatGPT in reproductive medicine: How promising and competent as Dr. Jekyll?

The rapid transformation of the field of reproductive medicine, characterized by complex methodologies and intricate ethical dilemmas, necessitates a continuous effort to enhance understanding and develop innovative strategies to improve patient-centered care (Wilkinson et al., 2018[6]). Within this *milieu*, ChatGPT materializes as an ostensibly propitious instrument for reproductive specialists, attributable to its voluminous repository of scholarly dissertations and the faculty to engender contextually germane ripostes (Grünebaum et al., 2023[3]). The extent to which ChatGPT can be effectively utilized as a reliable resource for medical practitioners is a subject of ongoing debate. This is primarily due to its tendency to fluctuate between providing valuable, informed knowledge akin to the wisdom of Dr. Jekyll, and disseminating potentially harmful misinformation reminiscent of the character Mr. Hyde. This inconsistency, if not attended, may raise significant concerns and apprehensions among professionals in the medical field.

On the one hand, remarkable aptitude of ChatGPT for the dissection and amalgamation of profuse data sets serves as an indispensable instrument in accelerating the procurement of pertinent knowledge from the immense repository of scholarly literature (Chavez et al., 2023[2]). Being conversant with the cutting-edge developments in assisted reproductive technologies (ART), such as *in vitro* fertilization (IVF) and intracytoplasmic sperm injection (ICSI), this sophisticated language model empowers reproductive experts to more proficiently assess the success rates of various treatments, consequently honing their clinical acumen and decision-making prowess. Moreover, through the adept application of natural language processing algorithms, ChatGPT exhibits a remarkable ability to demystify abstruse scientific principles and nurture patient comprehension. In doing so, it engenders a conducive environment for securing informed consent and reinforcing patient autonomy, thus fostering a more collaborative and transparent rapport between medical practitioners and their clientele (Kung et al., 2023[4]).

In the contemporary realm of reproductive medicine, the implementation of sophisticated AI system of ChatGPT plays an instrumental role in augmenting clinical acumen and facilitating efficacious therapeutic interventions. By leveraging the GPT-4 architecture, ChatGPT demonstrates an unprecedented capacity to assimilate, analyze, and interpret voluminous quantities of data, thereby contributing substantially to the advancement of these interrelated disciplines. ChatGPT functions as a powerful adjunct to clinical decision-making processes. By employing advanced machine learning algorithms, this AI-driven platform facilitates the rapid and accurate diagnosis of multifarious reproductive disorders, thereby expediting the implementation of targeted, evidence-based therapies. Additionally, the ability of ChatGPT to systematically parse through the ever-expanding corpus of medical literature serves to keep healthcare practitioners apprised of emerging therapeutic modalities and novel diagnostic techniques. In infertility management, proficiency of ChatGPT in discerning subtle patterns in complex datasets enables it to aid clinicians in the identification of underlying etiological factors contributing to suboptimal reproductive outcomes. Moreover, the capacity of AI system for sophisticated statistical analyses bolsters the development and refinement of personalized treatment regimens, thereby enhancing the likelihood of successful conception and pregnancy maintenance. In obstetrics and gynecology practice, aptitude of ChatGPT for processing a plethora of patient-specific variables, such as demographic data, medical histories, and imaging studies, allows for the provision of tailored care that addresses the unique needs of individual patients. Furthermore, this cutting-edge AI platform assists in the prevention, early detection, and management of obstetric and gynecologic complications, fostering the delivery of safe, effective, and patient-centered care. Moreover, employment of ChatGPT in reproductive biology research has engendered a renaissance in the field, as its capacity to process vast quantities of data expedites the discovery of novel molecular mechanisms underpinning reproductive function and dysfunction. In so doing, ChatGPT catalyzes the development of innovative therapeutic strategies and pharmacological agents, ultimately bolstering the armamentarium of clinicians specializing in reproductive medicine, infertility management, obstetrics, and gynecology practice. Thus, the integration of ChatGPT into the domains of reproductive medicine serves to propel these disciplines into a new era of data-driven, personalized, and patient-centered care. By harnessing the power of advanced AI systems such as ChatGPT, healthcare practitioners and researchers alike stand poised to effectuate significant improvements in reproductive health outcomes, thereby promoting the well-being of countless individuals worldwide.

### ChatGPT in reproductive medicine: instincts of Mr. Hyde?

Contrarily, the inherent limitations present within the artificial intelligence framework of ChatGPT make it susceptible to the spread of biased, incorrect, or outdated information. The unique learning approach, which involves the combination of text corpora from various sources, enhances the possibility of incorporating false materials or perpetuating mistaken beliefs (Arif et al., 2023[1]). Moreover, the absence of an effective method for ongoing assessment of knowledge accuracy intensifies concerns regarding the reliability of ChatGPT in the dynamic and ethically sensitive field of reproductive biomedicine (Grünebaum et al., 2023[3]).

The multifarious ethical consequences emerging from the deployment of ChatGPT in the realm of reproductive medicine necessitate scrupulous examination. The unintentional spread of incorrect information by this artificial intelligence system may potentially violate fundamental bioethical principles, including those of beneficence and non-maleficence. This could occur because the distribution of inaccurate advice may lead to less-than-optimal treatment results or inadvertently cause harm through medically induced errors (Wang et al., 2019[5]). In addition, the possibility of unintentional exposure or misuse of sensitive patient information during engagements with the AI system raises concerns about the maintenance of patient confidentiality and privacy. This not only amplifies the risk associated with discriminatory practices but also enhances the potential for the imposition of stigmatizing judgments or insinuations (Wang et al., 2019[5]).

### The debate goes on

In view of the aforementioned discussions, it is of utmost importance to exercise practical discretion in assimilating ChatGPT within the domain of reproductive medicine practice. Meticulous validation investigations ought to be executed to determine the precision, dependability, and pragmatic applicability of the artificial intelligence system in question (Chavez et al., 2023[2]). Furthermore, continuous monitoring and assessment of the performance of ChatGPT, in conjunction with the institution of a comprehensive structure for the ethical administration of AI in the medical field, are indispensable for safeguarding patient well-being and maintaining the principles that underpin the medical profession (Grünebaum et al., 2023[3]).

In summary, the incorporation of ChatGPT in the realm of reproductive medicine unveils both alluring prospects and imposing obstacles. As we endeavor to exploit the capabilities of AI to enhance patient treatment and outcomes, it is crucial to remain alert to the prospective hazards of this incipient technology, while upholding the inviolable tenets of medical practice. Only through such vigilance can we be assured that ChatGPT will adopt the disposition of a beneficent Dr. Jekyll, rather than an iniquitous Mr. Hyde.

## Declaration

### Ethical statement

Not applicable.

### Conflicts of interest

The authors declare no conflicts of interest.

### Acknowledgments

Not applicable.

## Supplementary Material

Supplementary information
