# Supplementary material for: ChatGPT guidance for reproductive specialists: Dr. Jekyll or Mr. Hyde?
Source: EXCLI J. 2023 Aug 29;22:911–4. doi: 10.17179/excli2023-6120 (PMC10502200; doi:10.17179/excli2023-6120)
Supplement: Supplementary information [file EXCLI-22-911-s-001.pdf]

EXCLI Journal 2023;22:911-914 – ISSN 1611-2156
Received: April 20, 2023, accepted: August 26, 2023, published: August 29, 2023

Supplementary information to:

Letter to the editor:

CHATGPT GUIDANCE FOR REPRODUCTIVE SPECIALISTS: DR. JEKYLL OR MR. HYDE?

Pallav Sengupta[1*], Sulagna Dutta[2]

[1] Department of Biomedical Sciences, College of Medicine, Gulf Medical University, Ajman, UAE
[2] School of Medical Sciences, Bharath Institute of Higher Education and Research (BIHER), Tamil Nadu, India

* **Corresponding author:** Pallav Sengupta, PhD, Department of Biomedical Sciences, College of Medicine, Gulf Medical University Ajman, UAE. Phone: +971503083217; E-mail: pallav_cu@yahoo.com

https://dx.doi.org/10.17179/excli2023-6120

This is an Open Access article distributed under the terms of the Creative Commons Attribution License (http://creativecommons.org/licenses/by/4.0/).

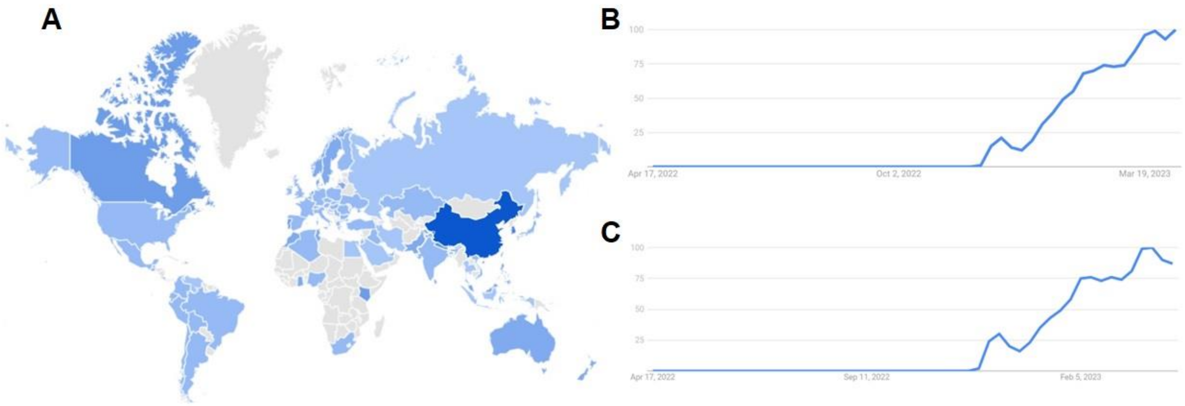

Supplementary Figure 1: Temporal progression of global ChatGPT utilization from April 2022 to April 2023, delineated across **(A)** healthcare sectors and **(B)** scientific domains **(C)**. An intensification in the usage trend is represented through a gradient of blue hues, with countries exhibiting minimal utilization manifested in light blue, progressing to countries with maximal utilization in dark blue **(A)**. Regions depicted in gray signify an absence of data pertaining to ChatGPT use within healthcare and science (Data Source: https://trends.google.com/trends/).
